# The Forebrain-Specific Overexpression of Acid Sphingomyelinase Induces Depressive-Like Symptoms in Mice

**DOI:** 10.3390/cells9051244

**Published:** 2020-05-18

**Authors:** Iulia Zoicas, Fabian Schumacher, Burkhard Kleuser, Martin Reichel, Erich Gulbins, Anna Fejtova, Johannes Kornhuber, Cosima Rhein

**Affiliations:** 1 Department of Psychiatry and Psychotherapy, Friedrich-Alexander Universität Erlangen-Nürnberg, 91054 Erlangen, Germany; iulia.zoicas@uk-erlangen.de (I.Z.); martin.reichel@charite.de (M.R.); anna.fejtova@uk-erlangen.de (A.F.); johannes.kornhuber@uk-erlangen.de (J.K.); 2 Department of Toxicology, University of Potsdam, 14558 Nuthetal, Germany; fabian.schumacher@uni-potsdam.de (F.S.); kleuser@uni-potsdam.de (B.K.); 3 Department of Molecular Biology, University of Duisburg-Essen, 45147 Essen, Germany; erich.gulbins@uni-due.de; 4 Department of Psychosomatic Medicine and Psychotherapy, Friedrich-Alexander Universität Erlangen-Nürnberg, 91054 Erlangen, Germany

**Keywords:** *Smpd1*, acid sphingomyelinase, forebrain, depressive-like behavior, anxiety-like behavior, ceramide

## Abstract

Human and murine studies identified the lysosomal enzyme acid sphingomyelinase (ASM) as a target for antidepressant therapy and revealed its role in the pathophysiology of major depression. In this study, we generated a mouse model with overexpression of Asm (Asm-tg^fb^) that is restricted to the forebrain to rule out any systemic effects of Asm overexpression on depressive-like symptoms. The increase in Asm activity was higher in male Asm-tg^fb^ mice than in female Asm-tg^fb^ mice due to the breeding strategy, which allows for the generation of wild-type littermates as appropriate controls. Asm overexpression in the forebrain of male mice resulted in a depressive-like phenotype, whereas in female mice, Asm overexpression resulted in a social anxiogenic-like phenotype. Ceramides in male Asm-tg^fb^ mice were elevated specifically in the dorsal hippocampus. mRNA expression analyses indicated that the increase in Asm activity affected other ceramide-generating pathways, which might help to balance ceramide levels in cortical brain regions. This forebrain-specific mouse model offers a novel tool for dissecting the molecular mechanisms that play a role in the pathophysiology of major depression.

## 1. Introduction

Major depressive disorder (MDD) is a severe and chronic mood disorder with a lifetime prevalence of more than 10% [1]. Key symptoms of MDD are a depressed mood and loss of interest, anhedonia, feelings of worthlessness, weight loss, and insomnia. Although MDD is a very common disorder, its pathogenesis is still unclear. The acid sphingomyelinase (ASM)/ceramide system was recently implicated in the pathogenesis of MDD [2]. ASM (human; murine: Asm) is a lysosomal glycoprotein that catalyzes the hydrolysis of sphingomyelin into ceramide and phosphorylcholine [3]. Ceramide is generated by the hydrolysis of sphingomyelin through the activity of ASM, neutral sphingomyelinase (NSM), or alkaline sphingomyelinase depending on the optimum pH of the enzyme [4]. Ceramide can also be generated by de novo synthesis [5], by the degradation of complex (gluco)sphingolipids [6] or through a salvage pathway involving reacylation of the degradation product sphingosine [7]. Several studies have reported altered sphingomyelin and ceramide metabolism in MDD, which increased ASM activity in peripheral blood mononuclear cells of patients experiencing a major depressive episode [8]. Similarly, plasma levels of several ceramide species, including Cer16:0, Cer18:0, Cer20:0, Cer24:1, and Cer26:1 but not Cer22:0 or Cer24:0, were increased in patients experiencing a major depressive episode during the past 2 years [9]. Higher plasma ceramide Cer16:0, Cer18:0, Cer20:0, Cer22:0, Cer24:0, and Cer24:1 levels were also observed in patients with MDD and bipolar disorder [10], and higher plasma levels of ceramide Cer16:0 and Cer18:0 and sphingomyelin SM18:1 were associated with the increased severity of depression symptoms in patients with coronary artery disease [11]. In contrast, plasma sphingomyelin SM26:1 [12], SM21:0 and SM21:1 [13] levels were decreased in MDD patients, and the SM23:1/SM16:0 ratio was negatively correlated with the severity of depressive symptoms in a Dutch family [14].

Similar deregulation of sphingolipid metabolism was found in rodent models of depression. For example, transgenic mice overexpressing Asm (Asm-tg) throughout the body showed an increased serum and hippocampal Asm activity and an increased hippocampal ceramide concentration, which was associated with a depressive- and anxiogenic-like phenotype in both social and nonsocial contexts [2,15,16]. Exposure to chronic unpredictable stress, a model that induces a depressive-like and anxiogenic-like phenotype [17], increased the levels of ceramide Cer16:0, Cer16:1, Cer18:1, Cer22:1, and Cer26:1 but not Cer18:0, Cer20:0, Cer20:1, Cer22:0, Cer24:0, Cer24:1, and Cer26:0 in the hippocampus and frontal cortex but not in the amygdala or cerebellum in mice. In contrast, the levels of sphingomyelin SM16:0, SM20:0, SM22:0, SM24:0, and SM26:0 but not SM18:0, SM18:1, SM24:1, and SM26:1 were reduced by chronic unpredictable stress [18]. Chronic administration of corticosterone, which is known to induce a depressive-like and anxiogenic-like phenotype [19], also increased ceramide Cer22:1 levels in the dorsal hippocampus and ceramide Cer20:0, Cer22:1, Cer24:1, Cer26:0 and Cer26:1 levels in the ventral hippocampus. Sphingomyelin SM16:0, SM18:0, SM18:1, SM20:0, SM22:0, SM24:0, SM24:1, SM26:0, and SM26:1 and ceramide Cer16:0, Cer16:1, Cer18:0, Cer18:1, Cer20:1, Cer22:0, and Cer24:0, however, were not altered by chronic corticosterone administration [20], suggesting that specific stressors might alter sphingolipid metabolism in a different way. The direct involvement of ceramide in the pathogenesis of depression was demonstrated in naïve mice, which developed a depressive-like phenotype after infusion of ceramide Cer16 but not Cer8 or Cer20 into the dorsal hippocampus [2,21]. Interestingly, Cer16 induced a predominantly nonsocial anxiogenic-like phenotype when infused into the basolateral amygdala, suggesting that ceramides alter depressive-like and anxiety-like behavior in a brain region- and ceramide species-specific way [21].

Understanding the role of the ASM/ceramide system in the pathogenesis of MDD might prove to be relevant for the development of an optimized treatment for MDD. The constitutive Asm-tg mouse model is an important tool for investigating the effects of Asm overexpression in the absence of a stressor-specific bias; however, one cannot exclude the effects of a systemic phenotype. Here, we report the generation and characterization of a conditional transgenic mouse model in which the expression of Asm is restricted to the forebrain (Asm-tg^fb^). Restriction to the forebrain is possible via the Emx1-cre mouse strain [22], a widely used strain to generate conditional transgenic mouse models [23]. *Emx1* encodes a transcription factor and is expressed in the developing forebrain [24], specifically in the excitatory neurons and astrocytes [22]. Asm overexpression is therefore restricted, thereby excluding the influence of any systemic phenotype.

## 2. Materials and Methods

### 2.1. Animals

Male and female mice (12 weeks old) overexpressing Asm in the forebrain (Asm-tg^fb^) were used in this study. These mice were generated by crossing female Asm-tg mice [2] with male Emx1IREScre homozygous mice, which possess the IRESCre recombinase-encoding sequence in the 3´ untranslated region of the Emx1 gene. IRESCre recombinase drives the expression of Cre recombinase starting on embryonic day 10.5, and this expression is restricted to the forebrain [22]. The Asm transgene is located on the X-chromosome. Therefore, the resulting female Asm-tg^fb^ mice were heterozygous, while males were hemizygous for the transgene. Male and female WT and Asm-tg^fb^ mice were individually housed for one week before the experiments started and remained so throughout the experiments. Mice were kept under standard laboratory conditions (12:12 light/dark cycle, lights on at 06:00 h, 22 °C, 60% humidity, with food and water *ad libitum*). Experiments were performed during the light phase between 09:00 and 14:00 in accordance with the recommendations in the Guide for the Care and Use of Laboratory Animals of the Government of Unterfranken and the Guidelines of the National Institutes of Health. All efforts were made to minimize animal suffering and to reduce the number of animals used.

### 2.2. Experimental Overview

In the first experiment, we assessed the behavior of Asm-tg^fb^ mice in comparison with that of WT mice. After one week of being housed individually, the social anxiety-like behavior of mice was tested in the social preference-avoidance test (SPAT). Four days later, the depressive-like behavior of mice was tested in the novelty-suppressed feeding (NSF) paradigm. Twenty-four hours later, mice were rapidly killed under CO_2_ anesthesia, and the blood and brains were collected for further analysis. Blood was collected through cardiac puncture and centrifuged for 10 min at 4 °C and 2000 rpm. The serum was extracted and stored at −80 °C until it was assayed. Brains were removed, snap-frozen and stored at −80 °C. Several regions in the forebrain (i.e., frontal cortex, dorsal striatum, septum, amygdala, hypothalamus, dorsal hippocampus, and ventral hippocampus), midbrain (ventral mesencephalon) and hindbrain (cerebellum) were dissected from coronal brain slices as described in previous studies [15,21]. In one hemisphere (counterbalanced between mice), we analyzed Asm activity in all dissected brain regions. In the frontal cortex, ventral hippocampus and dorsal hippocampus of the second hemisphere, we quantified several sphingolipids, including the ceramide species Cer16:0, Cer18:0, Cer20:0, Cer22:0, Cer24:0, and Cer24:1, the sphingomyelin species SM16:0, SM18:0, SM20:0, SM22:0, SM24:0, and SM24:1, sphingosine and sphingosine-1-phosphate (S1P).

In a separate experiment, we collected brains from male Asm-tg^fb^ and WT mice, which were snap-frozen and stored at −80 °C. The frontal cortex and total hippocampus were dissected from coronal brain slices. We isolated RNA and performed quantitative real-time PCR (qPCR) analysis to investigate the expression of *Smpd1* mRNA encoding Asm and the expression of mRNAs encoding a variety of enzymes involved in sphingolipid metabolism, including neutral sphingomyelinase (*Smpd3*), glucosylceramidase (*Gba2*) and sphingosine-1-phosphate lyase (*Sgpl1*).

### 2.3. Social Preference-Avoidance Test (SPAT)

The social anxiety-like behavior of mice was tested in the SPAT as previously described [21]. Mice were placed in a novel arena (42 × 24 × 35 cm), and after a 30-s habituation period, an empty wire mesh cage (7 × 7 × 6 cm) was placed near one of the short walls. After 2.5 min, the empty cage was replaced by an identical cage containing an unfamiliar age-, weight- and sex-matched mouse for an additional 2.5 min. The test was recorded and analyzed using JWatcher (V 1.0, Macquarie University and UCLA). An increase in the investigation time directed towards the mouse versus the empty cage indicated social preference and, thus, a lack of social anxiety. A decrease in the investigation time directed towards the mouse indicated social avoidance and, thus, a social anxiogenic-like phenotype.

### 2.4. Novelty-Suppressed Feeding (NSF) Paradigm

The depressive-like behavior of mice was tested in the NSF paradigm as previously described [16,21]. Mice were food-deprived for 24 h prior to testing with unlimited access to fluids. Mice were placed in a novel arena (50 × 50 × 50 cm) with their head facing one of the corners. Immediately afterward, a single food pellet (ssniff Spezialdiäten GmbH, Soest, Germany) was placed in the center of the arena. The feeding latency, which was defined as biting the food pellet for longer than 3 s, was manually analyzed according to the videos. An increased feeding latency indicated a depressive-like phenotype.

### 2.5. Determination of Asm Activity In Vitro

Asm activity was determined in homogenates from several brain regions of the forebrain (i.e., frontal cortex, dorsal striatum, septum, amygdala, hypothalamus, dorsal hippocampus, and ventral hippocampus), midbrain (ventral mesencephalon) and hindbrain (cerebellum) and blood serum. For the preparation of brain homogenates, 10–20 mg pieces of tissue were homogenized in 0.5 mL sucrose lysis buffer (250 mM sucrose, 1 mM EDTA, and 0.2% Triton X—100) using a TissueLyser LT bead mill (Qiagen, Hilden, Germany). Raw lysates were centrifuged at ≥10.000× *g* at 4 °C for 10 min, and the supernatants were transferred to new tubes. The protein concentrations were determined using a bicinchoninic acid kit (Sigma, Darmstadt, Germany). For the determination of Asm activity, 1 µg of protein was incubated with 0.58 µM *N*—(4,4—difluoro—5,7—dimethyl—4—bora—3a,4a—diaza—s—indacene—3—dodecanoyl)—sphingosylphosphocholine (BODIPY® FL C_12_—sphingomyelin; D—7711; Life Technologies, Darmstadt, Germany) in a 50 µL reaction buffer (50 mM sodium acetate pH 5.0, 0.3 M NaCl, and 0.2% NP—40) for 2 h at 37 °C; after incubation, 3 µL of the reaction mixture was spotted on a silica gel 60 plate (Macherey-Nagel; Düren, Germany), and the spots of ceramide and sphingomyelin were separated by thin-layer chromatography using 99% ethyl acetate/1% acetic acid (v/v) as a solvent [25]. The intensities of the BODIPY-conjugated ceramide and sphingomyelin fractions were determined using a Typhoon Trio scanner (GE Healthcare, München, Germany) and quantified with QuantityOne software (BioRad, München, Germany).

### 2.6. Sphingolipid Quantification by Liquid Chromatography Tandem-Mass Spectrometry (LC-MS/MS)

Tissue from the frontal cortex and ventral and dorsal hippocampus was subjected to lipid extraction using 1.5 mL methanol/chloroform (2:1, v/v) [26]. The extraction solvent contained d_7_—sphingosine (d_7_—Sph), d_7_—sphingosine—1—phosphate (d_7_—S1P), ceramide C17:0 (Cer17:0) and sphingomyelin C16:0—d_31_ (SM16:0—d_31_) (all Avanti Polar Lipids, Alabaster, Alabama, USA) as internal standards. Sample analysis was carried out by liquid chromatography tandem-mass spectrometry (LC-MS/MS) using either a TQ 6490 mass spectrometer (for Sph and S1P) or a QTOF 6530 mass spectrometer (for Cer and SM species) (Agilent Technologies, Waldbronn, Germany) operating in the positive electrospray ionization mode (ESI+). The following selected reaction monitoring (SRM) transitions were used for quantification: *m/z* 300.3 → 282.3 for Sph, *m/z* 380.3 → 264.3 for S1P, *m/z* 307.3 → 289.3 for d_7_—Sph and *m/z* 387.3 → 271.3 for d_7_—S1P. The precursor ions of the Cer or SM species (which differed in their fatty acid chain lengths) were cleaved into the fragment ions corresponding to *m/z* 264.270 or *m/z* 184.074, respectively [27]. Quantification of the ceramide species Cer16:0, Cer18:0, Cer20:0, Cer22:0, Cer24:0, and Cer24:1, the sphingomyelin species SM16:0, SM18:0, SM20:0, SM22:0, SM24:0, and SM24:1, sphingosine and S1P was performed with MassHunter Software (Agilent Technologies, Waldbronn, Germany). The determined sphingolipid amounts were normalized to the actual protein content (determined by the Bradford assay) of the tissue homogenate used for lipid extraction. The used nomenclature of sphingolipids indicates the number of carbon atoms and double bonds of the fatty acid side chain. All sphingolipid species analyzed contain a d18:1 sphingosine backbone. For example, Cer16:0 has a fatty acid side chain length of 16 carbon atoms and no double bond.

### 2.7. Extraction of RNA and Synthesis of cDNA

Total RNA was isolated from cortical and hippocampal tissue (<30 mg) using a TissueLyser LT bead mill (Qiagen, Hilden, Germany) and peqGOLD Trifast reagent (Peqlab, Erlangen, Germany) according to the manufacturers’ instructions, which was followed by RNA purification performed with the Purelink RNA Kit from Thermo Scientific (Schwerte, Germany) according to the manufacturer’s protocol. RNA qualities and concentrations were assessed using a Nanodrop ND-1000 UV-Vis spectrophotometer. A total of 500 ng of RNA was transformed into cDNA using the Quanta cDNA Kit (Gaithersburg, MD, USA) according to the manufacturer’s protocol.

### 2.8. Quantitative PCR Analysis

Quantitative real-time PCR was performed using cDNA from cortical and hippocampal tissue using a LightCycler 480 real-time PCR system (Roche, Mannheim, Germany) in SYBR Green format. We analyzed the expression of the following genes, for which the primer sequences can be found in our earlier publication [28]: *Asah1, Asah2, Cerk, CerS1, CerS2, CerS3, CerS4, CerS5, CerS6, Galc, Gba, Gba2, Sgms1, Sgms2, Sgpl1, Smpd1, Smpd3, Sphk1, and Sphk2; Gapdh* was used as a reference gene. qPCR reactions contained 5 μL FastStart Essential DNA Green Master Mix (Roche, Mannheim, Germany), 0.5 μM of each primer (20 µM) and 2.5 μL diluted cDNA (corresponding to 12.5 ng RNA) in a total volume of 10 μL. The temperature profile used consisted of 95 °C for 5 min followed by 40 cycles of amplification (95 °C for 10 s, 60 °C for 20 s, and 72 °C for 30 s). The threshold cycles (Ct) were determined with the “second derivative maximum” method, and the relative mRNA expression levels were calculated with the 2^-ΔΔCt^ method [29] using LightCycler 480 software (release 1.5.0).

### 2.9. Statistical Analyses

Statistical analyses were performed using SPSS Statistics version 21. Statistical significance was determined using Student’s *t*-test and two-way ANOVA, followed by Bonferroni post-hoc analysis when appropriate. Statistical significance was set at *p* < 0.05.

## 3. Results

### 3.1. Asm-tg^fb^ Mice Show an Increase in the Expression of Smpd1 mRNA Encoding Asm

**Figure 1:**
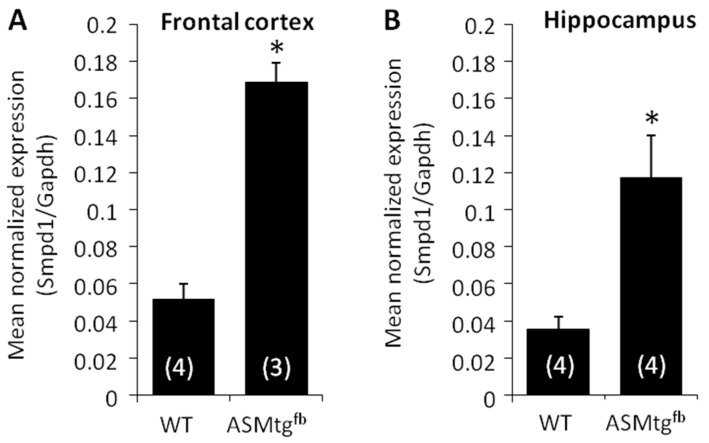
Asm-tg^fb^ mice show an increase in the mRNA expression of *Smpd1*, which encodes Asm. In both cortical and hippocampal tissues, male Asm-tg^fb^ mice showed a significant increase in *Smpd1* mRNA expression in comparison with WT mice. Data represent the means + SD, and numbers in parentheses indicate group sizes; * *p* < 0.05.

In the first analysis, we assessed *Smpd1* mRNA levels in male Asm-tg^fb^ mice to confirm our breeding strategy. In both cortical and hippocampal tissues, Asm-tg^fb^ mice showed a significant increase in *Smpd1* mRNA expression in comparison with WT mice (Figure 1A, frontal cortex, t(5) = −16.7; *p* < 0.001; Figure 1B, hippocampus, t(6) = −6.9; *p* < 0.001).

### 3.2. Asm-tg^fb^ Mice Show an Increase in Asm Activity in Forebrain-Related Brain Regions

To analyze whether increased *Smpd1* mRNA expression results in increased enzyme activity levels, we measured Asm activity in several regions of the forebrain (i.e., frontal cortex, dorsal striatum, septum, amygdala, hypothalamus, dorsal hippocampus, and ventral hippocampus), midbrain (ventral mesencephalon) and hindbrain (cerebellum) and the serum of male and female Asm-tg^fb^ mice. When compared with WT controls, both male and female Asm-tg^fb^ mice showed increased Asm activity in the dorsal striatum, dorsal hippocampus, ventral hippocampus, and amygdala. Male Asm-tg^fb^ mice also showed increased Asm activity in the frontal cortex, septum, and ventral mesencephalon. Female Asm-tg^fb^ mice showed increased Asm activity in the hypothalamus. Neither female nor male Asm-tg^fb^ mice showed increased Asm activity in the cerebellum or serum, confirming the regional specificity of ASM overexpression in the forebrain.

**Figure 2:**
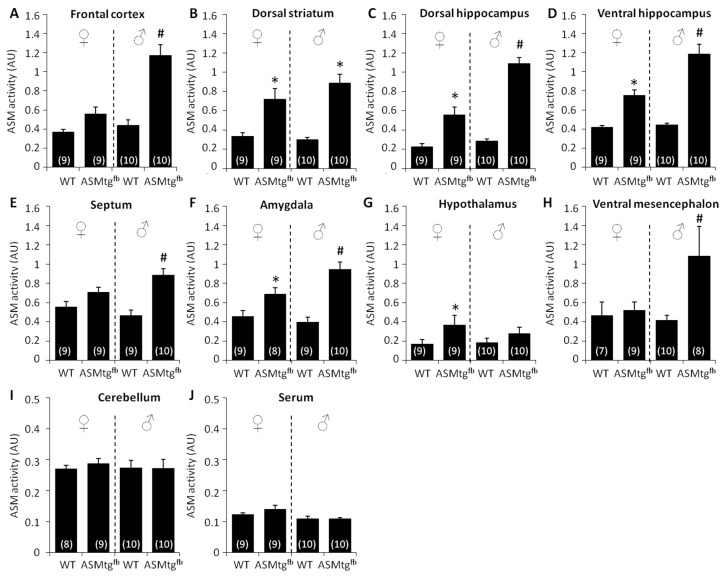
Brain Asm activity in WT and Asm-tg^fb^ mice. Asm activity was analyzed in nine different brain regions and blood serum for both males and females. In forebrain regions, Asm-tg^fb^ mice showed a significant increase in Asm activity levels compared with WT mice. Data represent the means + SEM, and numbers in parentheses indicate group sizes. * *p* < 0.05 versus same-sex WT; ^#^ *p* < 0.05 versus ♂ WT and ♀ Asm-tg^fb^.

Statistical results of increased Asm activity in male and female Asm-tg^fb^ mice: Dorsal striatum, Figure 2B, genotype effect F(1,34) = 34.2, *p* < 0.001; dorsal hippocampus, Figure 2C, genotype effect F(1,34) = 102.7, *p* < 0.001, sex × genotype effect F(1,34) = 16.7, *p* < 0.001; ventral hippocampus, Figure 2D, genotype effect F(1,34) = 72.1, *p* < 0.001, sex × genotype effect F(1,34) = 10.2, *p* = 0.003; amygdala, Figure 2F, genotype effect F(1,32) = 36.0, *p* < 0.001, sex × genotype effect F(1,32) = 5.71, *p* = 0.02. Statistical results of increased Asm activity only in male Asm-tg^fb^ mice: Frontal cortex, Figure 2A, genotype effect F(1,34) = 35.2; *p* < 0.001, sex × genotype effect F(1,34) = 11.9; p = 0.002); septum, Figure 2E, genotype effect F(1,33) = 13.3, *p* = 0.01, sex × genotype effect F(1,33) = 5.2, *p* = 0.03; ventral mesencephalon, Figure 2H, genotype effect F(1,30) = 4.67, p = 0.04. Statistical results of increased Asm activity only in female Asm-tg^fb^ mice: Hypothalamus, Figure 2G; genotype effect F(1,34) = 4.84; *p* = 0.04. No increase in Asm activity: Cerebellum, Figure 2I; genotype effect F(1,33) = 0.11; *p* = 0.74; serum, Figure 2J; genotype effect F(1,34) = 1.25; *p* = 0.27.

### 3.3. Male Asm-tg^fb^ Mice Show Increased Depressive-Like Behavior

**Figure 3:**
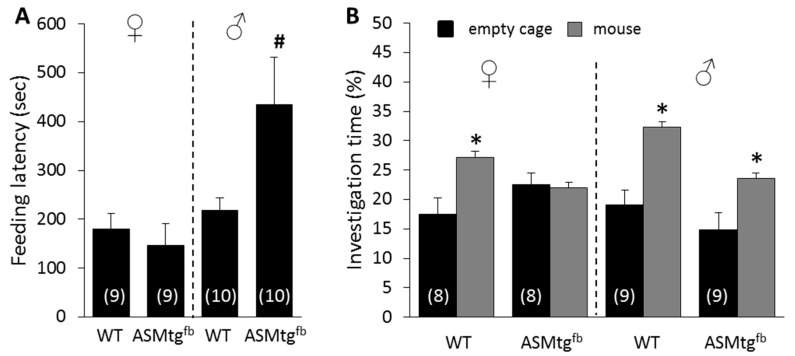
Asm overexpression alters depressive-like and social anxiety-like behavior in a sex-specific way. (**A**). Feeding latency, as an indicator of depressive-like behavior, was assessed in the novelty-suppressed feeding paradigm. Male Asm-tg^fb^ mice showed increased depressive-like behavior compared with WT mice. (**B**). The time of investigation of an unknown mouse compared with that of an empty cage, as an indicator of social anxiety-like behavior, was assessed in the social preference-avoidance test. Female Asm-tg^fb^ mice showed increased social anxiety-like behavior compared with WT mice. Data represent the means + SEM, and numbers in parentheses indicate group sizes. * *p* < 0.05 versus empty cage; ^#^ *p* < 0.05 versus ♂ WT and ♀ Asm-tg^fb^.

To investigate whether the increase in Asm activity induced a depressive-like phenotype, male and female Asm-tg^fb^ mice were tested in the NSF paradigm. Male but not female Asm-tg^fb^ mice showed an increase in the feeding latency after a fasting period of 24 h, reflecting an increase in depressive-like behavior (Figure 3A; sex × genotype effect F(1,34) = 5.37; *p* = 0.03).

### 3.4. Female Asm-tg^fb^ Mice Show Increased Social Anxiety-Like Behavior

To investigate whether the increase in Asm activity induced a social anxiogenic-like phenotype, male and female Asm-tg^fb^ mice were tested in the SPAT. Whereas WT females showed increased investigation of the mouse versus the empty cage during SPAT, which reflected a social preference and a lack of social anxiety, Asm-tg^fb^ females showed decreased investigation of the mouse, reflecting a social anxiogenic-like phenotype. In males, however, there was no effect of genotype (Figure 3B; group × stimulus effect F(3,60) = 2.86; *p* = 0.04). Although male Asm-tg^fb^ mice showed decreased investigation of the mouse when compared with male WT mice, this did not reach statistical significance (*p* = 0.10).

### 3.5. Male Asm-tg^fb^ Mice Show Changes in Ceramide Levels Only in the Hippocampus

To investigate whether the increase in ASM activity affects sphingolipid levels in brain areas relevant for MDD, tissue from the dorsal and ventral hippocampus and frontal cortex was used for lipidomic analysis. In the dorsal hippocampus, the percentage of Cer24:0 compared to total ceramides varied in a sex-specific manner, with only male ASM-tg^fb^ mice displaying higher Cer24:0 levels than WT males (sex × genotype effect F(1,34) = 4.5; *p* = 0.04). In the ventral hippocampus, male ASM-tg^fb^ mice showed a decreased percentage of Cer18:0 versus total ceramides compared to WT males (sex × genotype effect F(1,34) = 4.6; *p* = 0.04). Other sphingolipids were not changed in hippocampal tissue. In the frontal cortex, no effects of ASM overexpression on sphingolipids were detected.

### 3.6. Asm-tg^fb^ Mice Show Changes in the mRNA Expression of Other Sphingolipid-Metabolizing Enzymes

**Figure 4:**
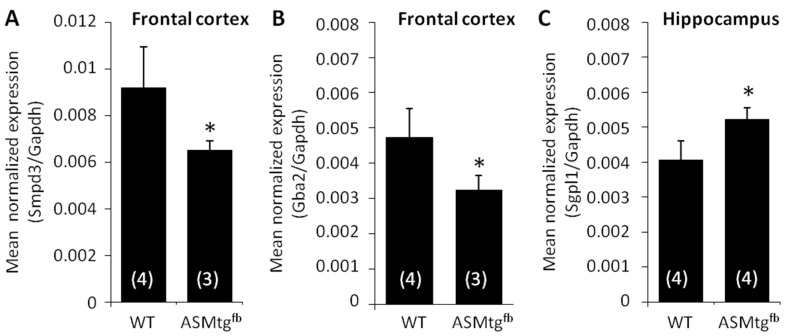
Asm-tg^fb^ mice show changes in mRNA expression of sphingolipid-metabolizing enzymes. In cortical tissue, male Asm-tg^fb^ mice showed a significant decrease in (**A**) *Smpd3* and (**B**) *Gba2* mRNA expression in comparison with WT mice. In hippocampal tissue, male Asm-tg^fb^ mice showed a significant increase in (**C**) *Sgpl1* mRNA expression in comparison with WT mice. Data represent the means + SD, and numbers in parentheses indicate group sizes; * *p* < 0.05.

To assess the cause of the relatively slight changes in ceramide levels despite the significant increase in Asm activity, we analyzed the mRNA expression of a variety of enzymes involved in sphingolipid metabolism. Interestingly, in cortical tissue of Asm-tg^fb^ mice, mRNA expression of neutral sphingomyelinase (Figure 4A; *Smpd3*; t(5) = 2.6; *p* = 0.049) and glucosylceramidase 2 (Figure 4B; *Gba2*; t(5) = 2.9; *p* = 0.04) were significantly decreased compared with that in WT mice. In the hippocampus, mRNA expression of sphingosine—1—phosphate lyase (*Sgpl1*) was significantly increased in Asm-tg^fb^ mice compared with that in WT mice (Figure 4C; t(6) = −3.6; *p* = 0.01). No changes were found in the expression of *Asah1, Asah2, Cerk, CerS1, CerS2, CerS3, CerS4, CerS5, CerS6, Galc, Gba, Sgms1, Sgms2, Sphk1*, and *Sphk2*.

## 4. Discussion

Our study characterizes a mouse model with increased Asm activity specifically in the forebrain. This increased Asm activity resulted in a depressive-like phenotype in males and a social anxiogenic-like phenotype in females. Compared with the Asm-tg model, which overexpresses Asm in the whole body [2,16], this conditional transgenic mouse model excludes the influence of a systemic phenotype.

The significant increase in *Smpd1* mRNA encoding Asm in cortical and hippocampal brain areas of Asm-tg^fb^ mice confirms the success of our breeding strategy. Similarly, ASM enzymatic activity was significantly increased in the forebrain as well as in areas with forebrain projections, including the frontal cortex, dorsal striatum, dorsal and ventral hippocampus, septum, amygdala, and hypothalamus. As expected, no increase in Asm activity in Asm-tg^fb^ mice was detected in the cerebellum, where no Cre is expressed in Emx1-cre strain mice [22], or in blood serum.

In most forebrain-related areas, the Asm activity levels of female Asm-tg^fb^ mice were lower than those of male Asm-tg^fb^ mice. In the frontal cortex and the septum, the increase in ASM activity levels in female ASM-tg^fb^ mice did not reach a significant level compared with that in WT female mice. Due to the location of the ASM transgene allele in the X chromosome, female ASM-tg^fb^ mice were heterozygous, while male ASM-tg^fb^ mice were hemizygous for the ASM transgene. In general, this results in the silencing of the respective transgene in female mice. Thus, the apparent sex differences in ASM activity levels might reflect the genetic situation more than sex differences per se. On the other hand, an exception is seen in the hypothalamus, where female Asm-tg^fb^ mice showed significantly higher Asm activity levels than male Asm-tg^fb^ mice. Given that the hypothalamus is a brain area highly relevant for several types of social behavior, including social anxiety [30], and that female but not male Asm-tg^fb^ mice showed a social anxiogenic-like phenotype, the increased Asm activity within the hypothalamus may contribute to social anxiety. Although female Asm-tg^fb^ mice showed a significant increase in Asm activity in the hippocampus, which is a brain area that was shown to be highly relevant for the pathology of MDD, they did not show a depressive-like phenotype. In contrast, male Asm-tg^fb^ mice showed clear changes in their behavior and displayed significant depressive-like behavior in the NSF test. Possibly, a very high threshold level of Asm activity in the hippocampus, as seen in male but not in female Asm-tg^fb^ mice, might be necessary to elicit changes in ceramide levels and depressive-like behavior. However, interestingly, male Asm-tg^fb^ mice showed an increase in Asm activity in the ventral mesencephalon, which might result from the close connections between forebrain regions and the mesencephalon. This might suggest the important role of the frontal cortex, septum, and ventral mesencephalon in the pathophysiology of MDD, given that male but not female Asm-tg^fb^ mice showed increased Asm activity within these brain areas. This points to a sex-specific effect, whereby increased Asm activity affects different circuits in female versus male Asm-tg^fb^ mice. The projections from the frontal cortex to the mesencephalon affecting the reward system could be essential for the control of emotional behavior in males and might be regulated sex-specifically. This could result in distinct subtypes of MDD for both sexes and might explain the different prevalence rates of MDD found in both sexes in human studies.

As ceramide is generated through the activity of Asm, a change in Asm activity levels is expected to alter ceramide levels, especially those in brain areas that are most relevant for depressive- and anxiety-like behavior, such as the hippocampus and frontal cortex. When looking at the effects of Asm overexpression on ceramides in the hippocampus, we found a sex- and brain region-specific effect. Male ASM-tg^fb^ mice displayed increased Cer24:0 levels in the dorsal hippocampus and decreased Cer18:0 levels in the ventral hippocampus compared with WT mice. This reflects the important role of the hippocampus in depressive- and social anxiety-like behavior. In particular, changes in ceramides in the dorsal hippocampus seem to be responsible for depressive-like behavior, which was also suggested by our earlier study [21]. In the hippocampus, the mRNA expression of sphingosine—1—phosphate lyase (*Sgpl1*) was significantly increased in Asm-tg^fb^ mice compared with that in WT mice. The enzyme S1P—lyase cleaves S1P to generate phosphoethanolamine and hexadecenal and plays an essential role in sphingolipid metabolism because this reaction cannot be reverted [31]. An increase in S1P—lyase would be associated with higher rates of irreversible cleavage of S1P. S1P is considered to be toxic in neurons since it induces stress in the endoplasmic reticulum and increases intracellular calcium currents [32,33,34]. Thus, neuronal cells might increase the expression of the *Sgpl1* gene, encoding S1P—lyase, to eliminate toxic S1P as a rescue mechanism. The increased ceramide levels, generated by increased Asm activity levels in Asm-tg^fb^ mice, could result in increased production of S1P, which is irreversibly cleaved by S1P—lyase and eliminated from the rheostat.

Lipidomic analyses revealed no significant changes in ceramides in the frontal cortex in male Asm-tg^fb^ mice showing a depressive-like phenotype. When we analyzed the mRNA expression of a variety of sphingolipid-metabolizing enzymes in the frontal cortex more closely, we found a significant decrease in the mRNA expression of neutral sphingomyelinase (*Smpd3*) and glucosylceramidase (*Gba2*) in Asm-tg^fb^ male mice in comparison with that in WT male mice. Neutral sphingomyelinase converts sphingomyelin into ceramide, but this mainly occurs at a neutral pH and at the plasma membrane; in contrast, ASM mainly generates ceramide at an acidic pH and in the lysosome [35]. Glucosylceramidase 2 converts complex glucosylceramides into ceramide and is located at or close to the cell surface [36]. Thus, given that the mRNA expression levels reflect the enzymatic activity, a decrease in *Smpd3* and *Gba2* expression would result in a decrease in ceramide levels. The overexpression of Asm in our mouse model, which should result in increased ceramide levels, might be counterbalanced by decreases in other enzymes that generate ceramide, which might explain why no changes in ceramide levels in the frontal cortex were observed in our analyses. The question remains whether the subcellular determination of ceramide localization could determine the differences in ceramide distribution in the frontal cortex of Asm-tg^fb^ mice.

Our results might provide novel insights into the complex regulation of the sphingolipid rheostat. Further studies should apply methods to determine the subcellular localization of ceramides to determine their specific roles. Sphingolipid metabolism is highly dynamic and well balanced [37]. In our mouse model, Asm was consistently overexpressed in the forebrain starting at an early developmental stage. The impact of ASM overexpression on ceramide species in different brain areas seems to reflect the complex mechanisms of sphingolipid metabolism.
